# Late Gadolinium Enhancement of the right ventricular myocardium: Is it really different from the left ?

**DOI:** 10.1186/1532-429X-10-20

**Published:** 2008-05-08

**Authors:** Lars Grosse-Wortmann, Christopher K Macgowan, Logi Vidarsson, Shi-Joon Yoo

**Affiliations:** 1 Section of Cardiac Imaging, Department of Diagnostic Imaging, The Hospital for Sick Children, The University of Toronto, Toronto, Canada; 2 Division of Cardiology, Department of Paediatrics, The Hospital for Sick Children, The University of Toronto, Toronto, Canada; 3 Department of Pediatric Cardiology, RWTH University of Aachen, Germany; 4 Department of Medical Biophysics, The Hospital for Sick Children, The University of Toronto, Toronto, Canada; 5 Department of Medical Imaging, The Hospital for Sick Children, The University of Toronto, Toronto, Canada

## Abstract

It has been suggested that, in late gadolinium enhancement, the signal of right ventricular myocardium is nulled at a shorter inversion time than the left. While we initially made the same observation, we believe that the difference is not real, but results from artifacts.

We present 7 cases as well as computer simulations to describe the nature of these artifacts and explain how they can create the impression of different inversion times for the right and left ventricle. At inversion times that are shorter than ideal for the myocardium a black rim can be seen at the border of the myocardium with blood on the inside and with fat on the outside. This is most likely a partial volume effect. The thin myocardium of the right ventricle is sandwiched between these black rims and, at a low spatial resolution, is no longer visible. In this case, the adjacent black rims may then be misinterpreted as myocardium. While black rims also occur on the left side, the myocardium is thicker and remains discernable as a separate layer. As a consequence, the optimal inversion time for the right ventricle only appears different from that for the left. In fact, in the presence of hypertrophy of the right ventricle or during systolic wall thickening we did not find a difference in inversion times between the left and right ventricle. We conclude that sufficient spatial resolution is important for adequate late gadolinium enhancement of the right ventricle.

## 1. Introduction

Late Gadolinium Enhancement (LGE) sequences have become a routine part of cardiovascular magnetic resonance (CMR) for ischemic heart disease, myocarditis and the cardiomyopathies [1-6]. In pediatric cardiology, LGE is used most often to look for fibrosis in suspected arrhythmogenic right ventricular dysplasia (ARVD)[7,8]. In this condition, compared to the findings in acute myocardial infarction, LGE is less pronounced and more diffuse. In the right ventricle (RV), even transmural fibrosis may only be < 2 mm thick[9]. Moreover, LGE is rare in pediatric ARVD patients and, if present, subtle[10]. For these reasons, an optimal contrast-to-noise ratio between normal and fibrotic myocardium is even more important than for the LV myocardium. In order to maximize the contrast between healthy and enhanced necrotic or fibrotic myocardium, the aim is to suppress the signal from normal myocardium. This is achieved by a non-selective 180° inversion pulse. After a certain time interval following the inversion pulse (TI), the signal intensity from a particular tissue is zero. The duration of TI depends on the type of tissue and the elapsed time after the administration of contrast[11]. TI of blood is shorter than that of viable myocardium, and longer than that of infarcted tissue, since the latter retains some gadolinium which shortens T1[11]. Desai and co-workers, as well as Amano et Kumazaki, suggested that the RV has a shorter optimal TI than the left ventricle (LV)[12,13]. While we have observed a similar apparent difference in TI between the RV and LV in patients with normally thin walled RVs, our clinical practice has convinced us that this discrepancy is merely suggested by an artifact. This report illustrates our observations in four representative clinical cases. We then tested our hypothetical explanation of the seemingly apparent but false differences between the two ventricles using a numerical model and in three more clinical cases.

## 2. Methods

### 2.1. Clinical Cases

All LGE sequences followed 10 to 20 minutes after a bolus of 0.2 mmol/kg of dimeglumine gadopentetate (Magnevist, Berlec Laboratoires, Quebec, Canada). Cases 1–4 were performed on a 1.5 Tesla scanner (Signa CV/*i*, General Electric Medical Systems, Milwaukee, MI, USA) using a 4-channel surface coil and a spoiled gradient refocused echo sequence, without phase sensitive reconstruction (PSIR) capabilities. The settings were as follows: Field of view (FOV) 34 – 40 cm, matrix 256 × 192, slice thickness 7.0–8.0 mm, minimum echo (TE) and repetition (TR) times (3.1 and 7.1 ms, respectively), flip angle 20°, bandwidth 31.25 kHz, number of excitations (averages, NEX) 2, views (lines) per segment (VPS) 24, trigger delay set to target diastole.

Cases 5, 6, 7 and 8 were scanned on a different magnet (1.5 Tesla, Avanto, Siemens Medical Solutions, Erlangen, Germany), using a 12-channel cardiac coil and a double inversion steady state free precession sequence (IR TrueFisp), with the following settings: In-plane resolution 1.5 mm by 3 mm for patient 5, 1.5 by 1.1 mm in diastole and 2.1 by 1.5 in systole for patient 6, 1.6 by 1.6 mm as well as 2.6 × 2.6 mm for patient 7 and 1.4 × 1.4 mm for patient 8, slice thickness 7 mm for patients 5 and 6, 10 mm for patient 7 and 7 as well as 10 mm for patient 8, TE 1.02 to 1.34 ms, TR 8.2 to 9.0 ms, NEX 2, VPS 65. In order to obtain the systolic image in patients 5 and 6 the data were acquired every three heart beats instead of every two in order to target systole early after the R spike. The temporal resolution was shortened to target a period of minimal cardiac motion at the end of systole by reducing the VPS to 40. TI was chosen to produce a black rim artefact in patient 5.

### 2.2. Computer Simulations

Assuming T1 times for gadolinium-containing blood, myocardium, and fat of 330 ms, 450 ms, and 300 ms, respectively, we generated a computer model of a ventricle in short axis with simulations of these three tissues from the inside to the outside. The virtual ventricle is surrounded by zero signal, mimicking the air-filled lungs. The simulations included varying TIs and in-plane resolutions.

## 3. Results

### 3.1. Clinical Cases

**Figure 1:**
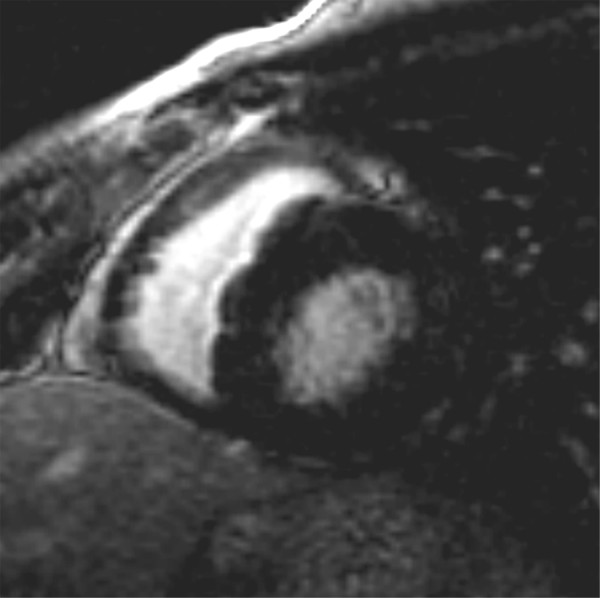
**The right ventricle is hypertrophied.** The signals from both the right and left ventricular myocardium are similarly suppressed at the same inversion time. Most trabeculations along the right ventricular free wall are grey, due to partial voluming with the blood, in contrast to a few larger trabeculations that show a black signal which is similar to that of the compact layer of the right and left ventricular myocardium.

Case 1 (Figure 1): 17 years old male adolescent with hypertrophic cardiomyopathy, involving the RV. Differences between the RV and LV at a given TI cannot be detected. The trabeculations and thinner parts of the RV free wall are grey, while the thicker parts of the RV free wall as well as the ventricular septum and LV free wall are black.

**Figure 2:**
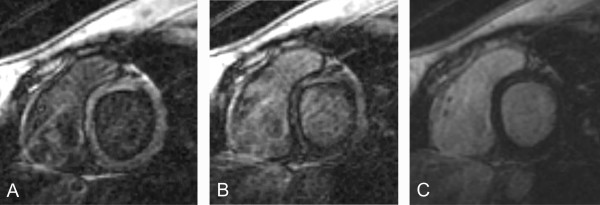
**Images A) to C) display late gadolinium enhancement images at inversion times between 210 ms, 230 ms, and 260 ms.** The window levels are optimized for each setting. In A) and B), a black rim of constant thickness is found, with varying signal intensities of the blood and the myocardium. At an inversion time of 260 ms, the myocardial signal is nulled and the rim has disappeared.

Case 2 (Figure 2): 17 years old girl with regional RV wall motion abnormalities and suspected ARVD. A black rim artifact can be seen at a suboptimal TI, but not at an optimal TI.

**Figure 3:**
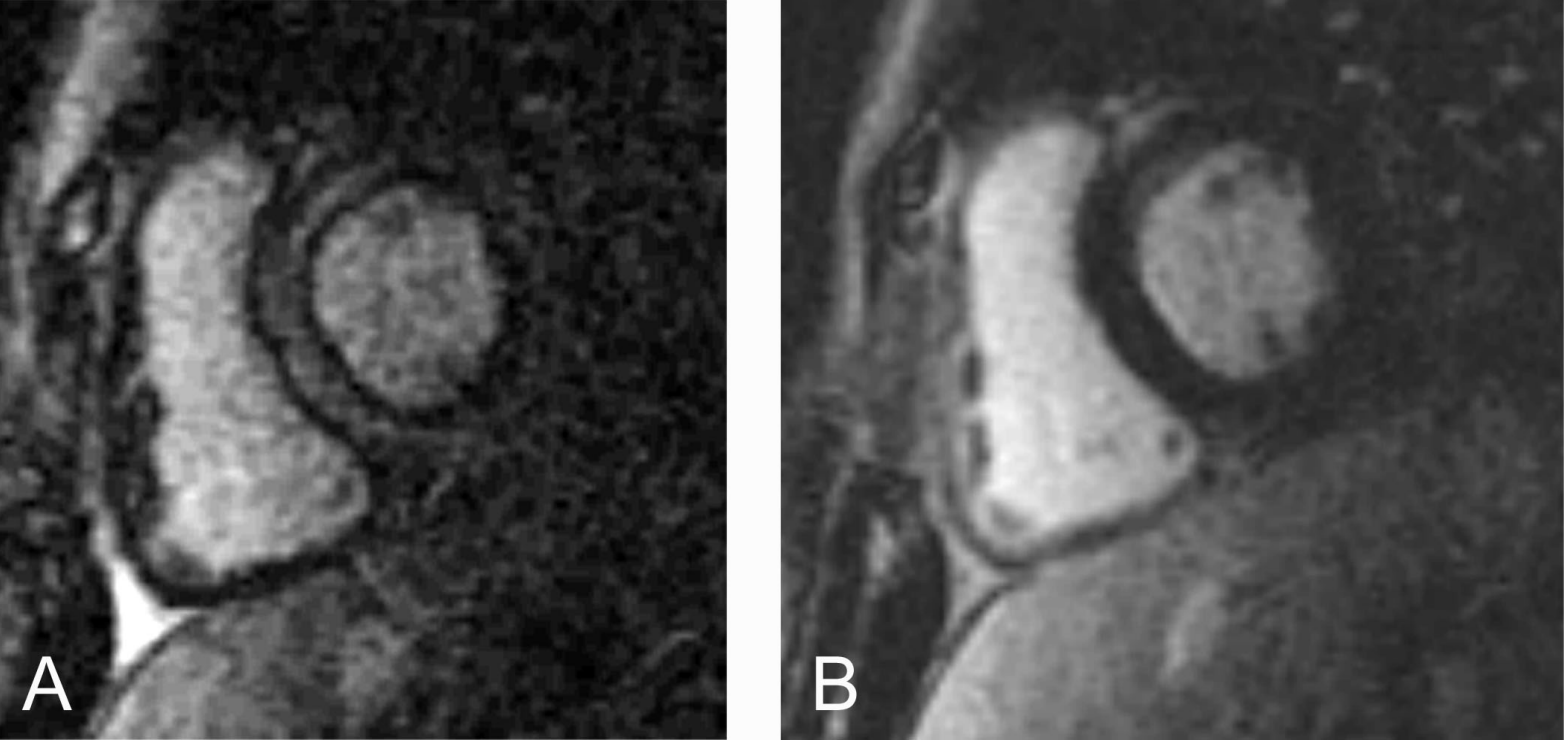
**Late gadolinium enhancement images were obtained with an inversion time of 220 ms (A) and an inversion time of 280 ms (B). **The myocardium of the left ventricular posterior wall and interventricular septum is incompletely suppressed in A) and properly suppressed in B). In A), the myocardium is demarcated from the blood and epicardial fat by black rims. Note that the trabeculations along the anterior free wall of the right ventricle are also outlined by black rims. The myocardium itself, however, is not nulled. In the anterior free wall, the muscular layer is not discernable from the black rims. As a result, the wall appears black.

Case 3 (Figure 3): 13 years old boy who underwent a CMR to rule out ARVD. Cine imaging showed a dilated and mildy hypocontractile RV. This example shows how the presence of a black rim affects the apparent thickness of the adjacent myocardium. In Figure 3A, large parts of the RV anterior free wall appear black while the LV myocardium is grey, suggesting different TIs for the two ventricles. The signal in the RV trabeculations, in contrast to the compact layer of the anterior free wall, is not black. In Figure 3B, the trabeculations appear nulled, as does the LV myocardium. Also, the size of the right ventricular trabeculations appear smaller in Figure 3B than in 3A because they are demarcated by the black rim in Figure 3A.

**Figure 4:**
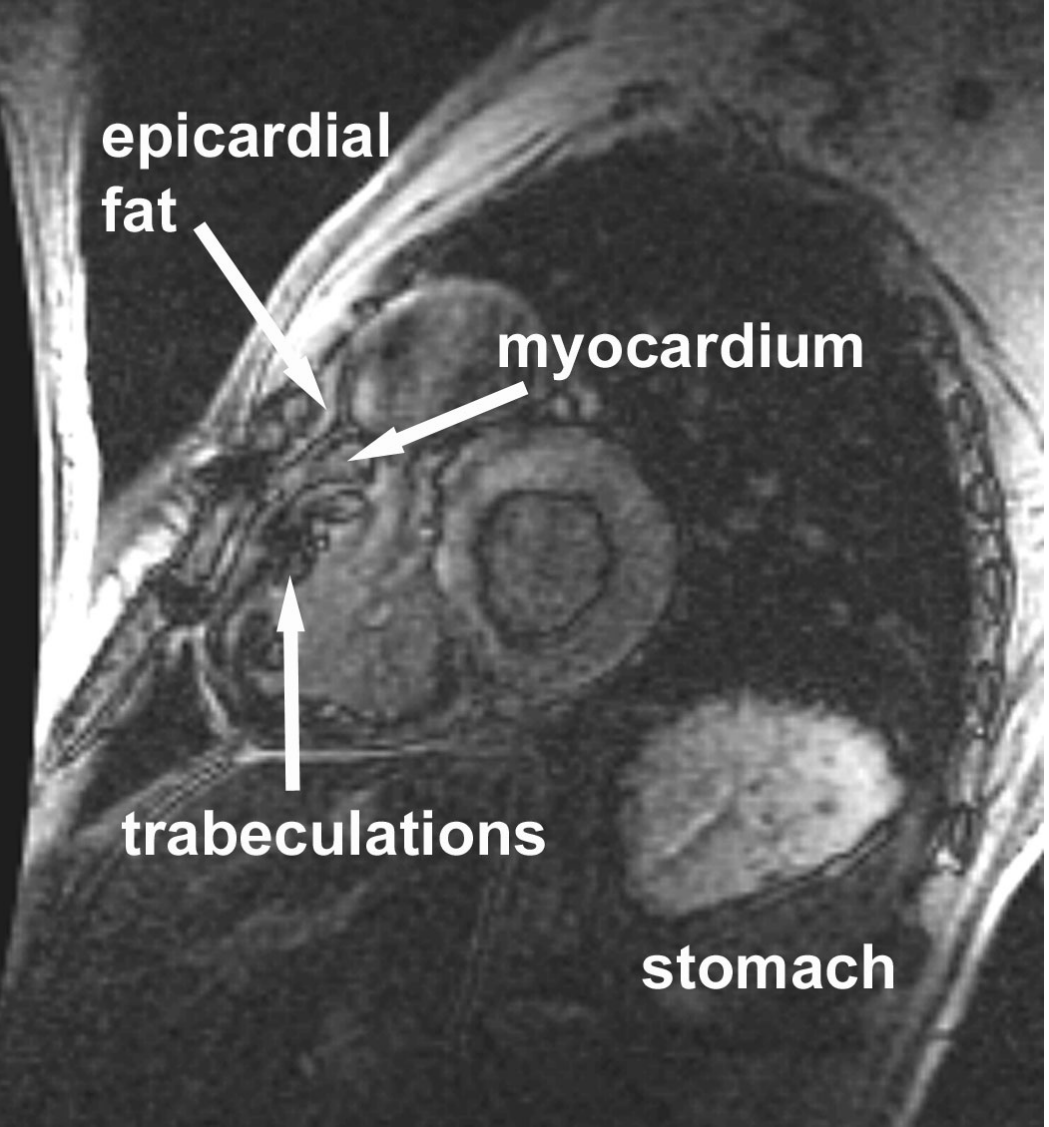
**The inversion time for this image was 200 ms. **The layers of the ventricular walls are delineated. Note the trabeculations, surrounded by black rims. A black rim also demarcates the outer border of the myocardium at the interface with the epicardial fat.

Case 4 (Figure 4): 17 years old male with repaired Tetralogy of Fallot. The RV is dilated and hypertrophied as a consequence of free pulmonary insufficiency. The myocardium is surrounded by a black rim on the luminal border and separated from the epicardial fat by another rim. A similar black line can also be observed outlining the gastric mucosa.

**Figure 5:**
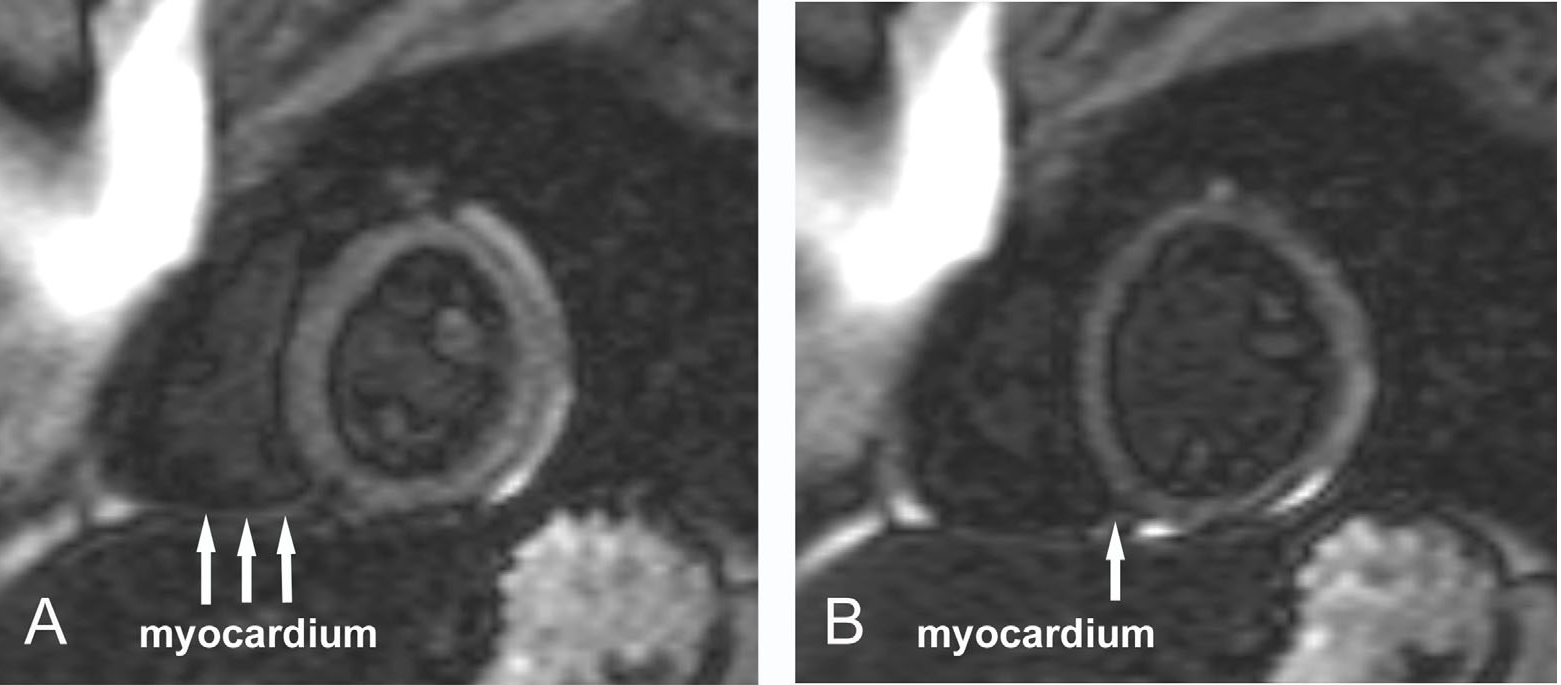
**Late gadolinium enhancement images in short axis.** During systole (A) the black rim and the myocardium are clearly discernable for the right ventricular inferior wall. In contrast, at end-diastole (B), when the myocardium is thinner, only a small part of it is discernable in the same region near the septum. A black rim outlines the thicker left ventricular myocardium in systole and diastole. The in-plane and through-plane spatial resolution of A) and B) is identical.

Case 5 (Figure 5): The 15 year old boy who was scanned for a family history of ARVD. Short axis plane images were obtained at mid-systole (Figure 5A) and end-diastole (Figure 5B). In systole, the RV myocardium is thicker than during diastole and can be traced throughout the inferior wall. At end-diastole, only the black rim can be appreciated.

**Figure 6:**
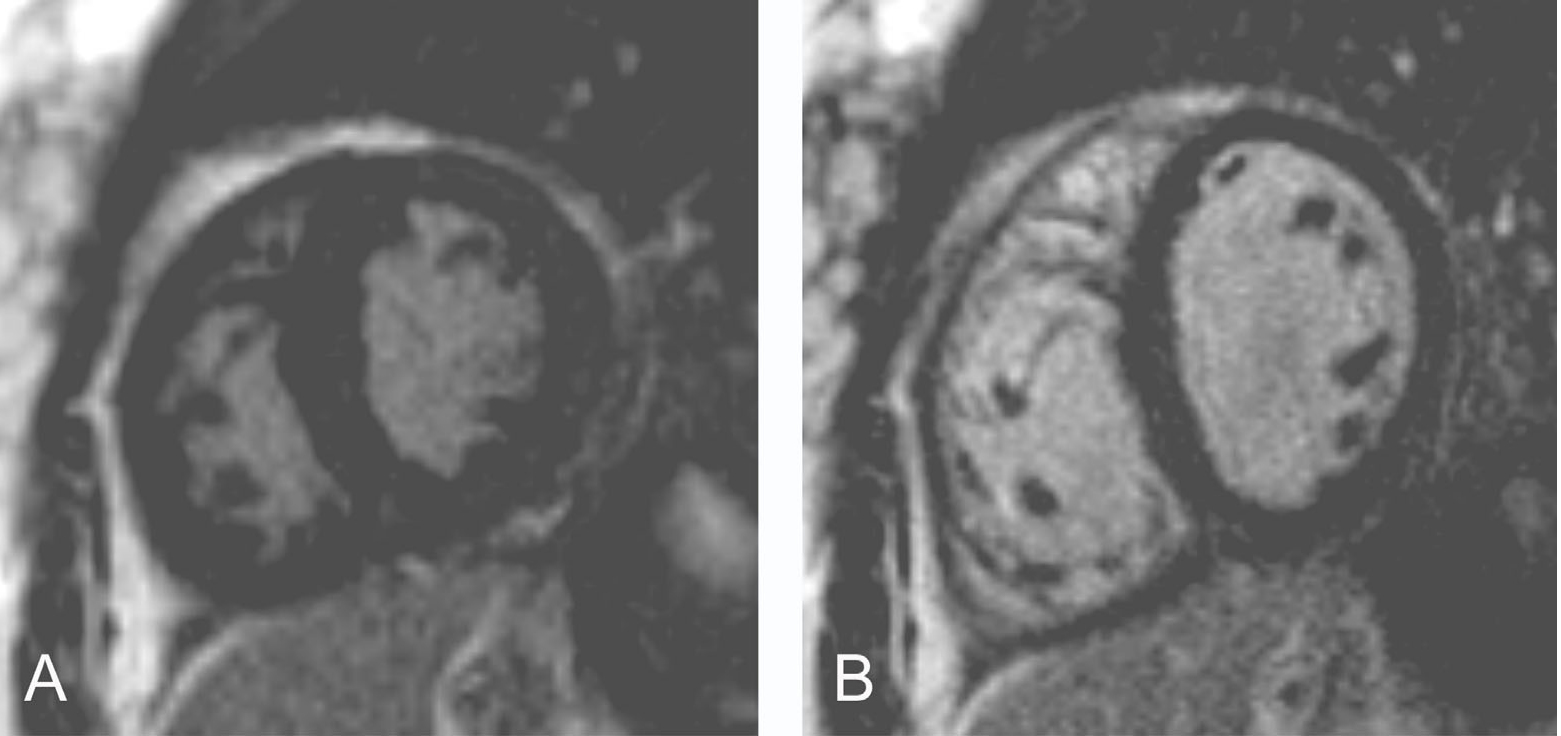
**Inversion recovery images in short axis using an inversion time to null the LV myocardial signal.** At end-diastole (Figure 6B) the RV inferior wall is black whereas the trabeculations appear grey and not adequately nulled. During systole (Figure 6A) the RV free wall thickens and compacts, clearly demonstrating an inversion time that is similar to the LV.

Case 6 (Figure 6): This 14 year old girl was born with transposition of the great arteries, ventricular septal defect and valvar pulmonary stenosis. She underwent a Rastelli operation (patch closure of the ventricular septal defect creating a tunnel from the LV to the aortic valve and insertion of a conduit between the RV and the main pulmonary artery). The RV is hypertrophied. The fact that the TI is similar for both ventricles is better appreciated in systole (Figure 6A) than in diastole (Figure 6B).

**Figure 7:**
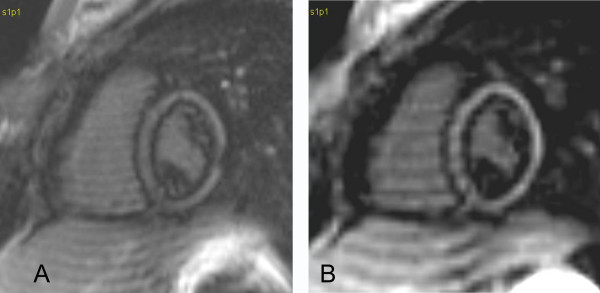
**Late gadolinium enhancement images with pixel sizes of 1.6 × 1.6 (A) and 2.6 × 2.6 mm (B). **The black rim increases with decreasing spatial resolution between A and B.

Case 7 (Figure 7): 17 year old girl who underwent a CMR to assess the reason for her dilated RV. She was found to have a sinus venosus atrial septal defect with partial anomalous pulmonary venous connection. At a constant slice thickness of 10 mm and a TI of 200 ms, the inplane resolution varied. The black rim increases in thickness with increasing pixel size (Figure 7).

**Figure 8:**
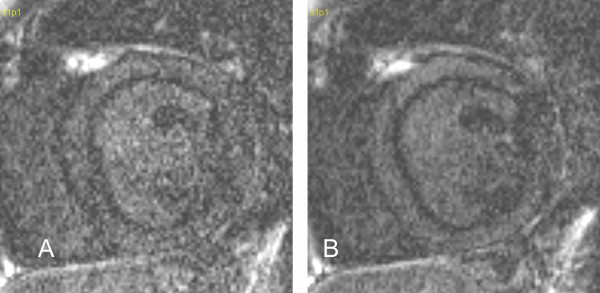
**Black rim artifacts in images with 7 mm (A) and 10 mm (B) thick slices that are otherwise identical. **The width of the rim remains unchanged between the two images.

Case 8 (Figure 8): When we obtained two consecutive images with slice thickness of 7 mm (Figure 8A) and 10 mm (Figure 8B) in this 9 year old boy with mixed aortic valve disease at a suboptimal TI the resulting black rim artifact was of similar thickness in both images.

### 3.2. Computer Simulations

**Figure 9:**
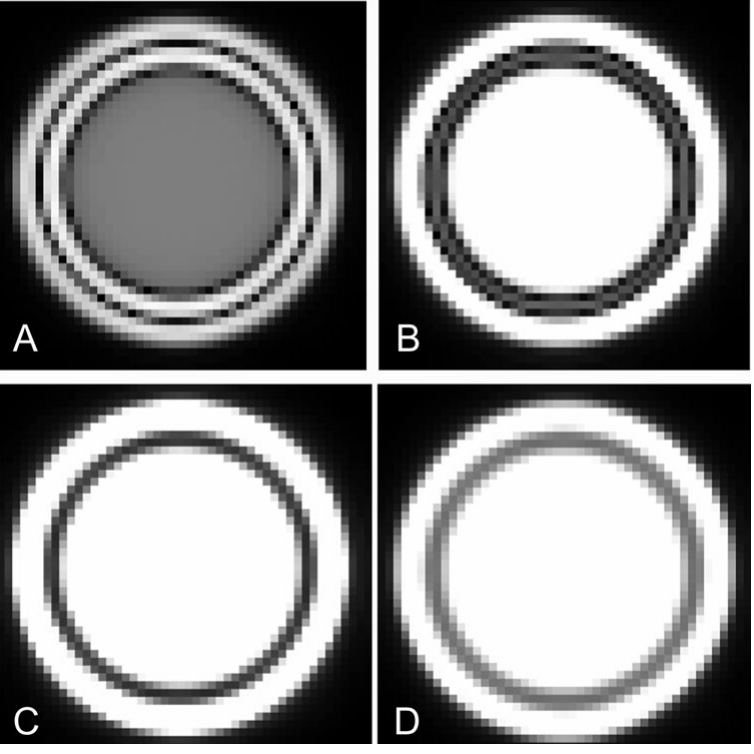
**Computer simulations of late gadolinium enhancement images of a ventricle at different inversion times: A) 250 ms, B) 280 ms, C) 310 ms, and D) 340 ms.** Pixel size is 1 mm^2^. The "myocardial" signal is best suppressed in C). At shorter inversion times (A + B), black rims can be seen the myocardium/blood as well as at the myocardium/epicardial fat interface, whereas they do not appear at ideal or longer than optimal inversion times (D).

**Figure 10:**
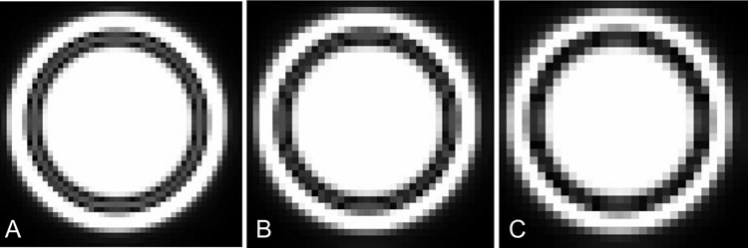
**Simulations of late gadolinium enhancement images at A) high (1 mm), B) intermediate (1.25 mm), and C) low (1.5 mm) spatial resolutions. **The simulated TI was 280 ms in all images. At all resolutions, the black rim artifact is one pixel thick and therefore increases in width with decreasing spatial resolution. In C), the right ventricular myocardium is sandwiched and non-discernable between the adjacent black rim artifacts. The blood/endocardium boundary is delineated best when it is parallel to the pixel sides.

The computed model exemplify the behavior of the black rim artifact at varying TIs (Figure 9) and spatial resolutions (Figure 10). See figure legends for details.

## 4. Discussion

It has been suggested that the TI for the RV myocardium is, truly or apparently, different from the LV myocardium[12,13]. Amano et al. speculated that differences in the myocardial tissue, in coronary blood flow or the denser RV conduction tissue as compared to the left side may be responsible for this discrepancy[13]. They thought it possible that the proximity of the RV to the receiver coil, although it does not affect TI, may cause more signal in the RV free wall as compared to the LV. They also recognized the possibility of partial volume effects as an explanation for a different TI for the RV. Remarkably, the majority of their patients (65%) had similar, rather than lower, TIs for the RV and the LV[13].

Using case examples and, for clarity, computer simulations, we showed that if the spatial resolution is sufficient to clearly visualize the thickness of the RV myocardium, then the TIs for the RV and LV myocardium are most likely similar. (Figures 1, 6, 9, 10). If this is the case, why was the normal RV wall completely not nulled and contains signal at the optimal TI for LV myocardium (Figures 1, 2C, 3B)? We hypothesize that in the images where the myocardial signal was optimally suppressed, the RV myocardium had some residual signal because of partial volume effects of the high signal of the blood on the thin RV myocardium. Case 6 (Figures 6A and 6B) convincingly illustrates this concept by detecting more signal in parts of the RV wall and the trabeculations when these are thinned during diastole using the optimal LV TI. At the same TI, but during systole when the RV muscle has compacted, there is no apparent difference of TI between the RV and the LV myocardium.

We also need to explain why the RV myocardium appears black at TI times that do not null the LV myocardial signal (Figure 3A). We conjecture that a black rim artifact at the inferface between the myocardium and blood has mistakenly been regarded as the RV myocardium, which has led to a wrong conclusion that the RV and LV myocardium have different optimum TI values. Our hypothesis is also supported by the fact that distinct black rims, similar to the one found in the LV, were observed not only outlining the RV free wall but also around the RV trabeculations in patients with hypertrophied RV (Figures 1, 4). In contrast to the thick RV free wall and trabeculations, the normal RV myocardium was not discernable in between the black rim artifacts because the RV wall was thin and the trabeculations are small (Figures 2, 3). The same is observed within the same patient during myocardial thickening in systole and thinning in diastole (Figures 5, 6). We strongly believe that the black rims, with a hardly discernable true myocardial layer in between may have wrongfully been perceived as nulled RV myocardium (Figures 3A).

The significance of this phenomenon for the detection of areas of fibrosis or ischemic damage in the RV wall is yet to be investigated. All vendors tailored their LGE sequences to target end-diastole because this is the time of the least cardiac motion at normal or only mildly elevated heart rates and because LGE is typically used in ischemic heart disease with clearly demarcated areas of infarction, mostly in the LV. LGE for myocardial infarction of the RV anterior wall remains to be proven useful and there is data to suggest that it is less sensitive than for the LV and the RV inferior wall [14-16]. In a study by LaRose et al. only 4 out of 25 patients with RV myocardial infarction and an ejection fraction of the RV < 40% demonstrated LGE of the RV[16]. The usefulness of LGE in children with suspected ARVD remains to be proven, likely in part due to the difficulty to detect subtle changes at early stages of the disease. We demonstrate that LGE imaging of the heart in systole is feasible (Figures 5A, 6A). It may be advantageous for discrete areas of fibrosis affecting the RV myocardium in ARVD and ischemic heart disease.

What causes the black rim? Dark rim artifacts in LGE imaging are well recognized[17,18] and have been attributed to susceptibility, motion, Gibbs ringing and partial volume effects[19]. Although all of the above are conceptually possible, susceptibility artifacts are typically found with high gadolinium concentrations, and Gibbs ringing is more pronounced in the spatial dimension encoded at lower spatial resolution, which is usually the phase-encoding direction. The dark rim artifacts in LGE imaging are remarkably regular and evenly distributed in all dimensions (Figures 2, 3, 4, 5, 7, 8, 9, 10). They most likely represent partial volume effects. The voxels at the myocardium/blood border contain both muscle and blood. After the inversion prepulse, the magnetization of these two tissues will recover, but at different rates depending on their respective T1-relaxation rates. Blood will typically recover more quickly, and, at some time after inversion will have a positive longitudinal magnetization while the myocardial magnetization is still partially inverted. When a second radiofrequency pulse, for example of 90°, is applied at that time, the magnetizations of the two tissues will both be tipped into the transverse plane but will be oriented at 180° with respect to each other. Because these signals originate from the same voxel, the two signals will destructively interfere with each other, producing signal loss manifested as a dark rim[17,18]. In principle, the larger the difference in tissue T1, the more distinct the artifact. Following this concept, the width of the black rim should be equal to the pixel size and therefore should decrease with increasing the in-plane spatial resolution (Figures 7, 10). The thickness and distinctness of the black rim artifact may also be affected by the slice thickness if the ventricular wall is not perpendicular to the imaging plane. The effect of partial voluming from increasing slice thickness in short axis cuts was minimal because the imaging planes are perpendicular to the ventricular wall and the relative composition of the voxel was unlikely to change dramatically (Figure 8). In addition, the fact that the black rim occurs exclusively with TIs that are shorter than ideal for myocardium and longer than ideal for blood supports its perception as a partial volume error. This also serves to strengthen our hypothesis that this artifact is the reason for the reported difference in TI between LV and RV myocardium, as the RV TIs were also stated to be shorter as compared to the LV[12]. The black rim can appear in voxels that do not contain equal amounts of both blood and myocardium, as long as the chosen TI lies between the optimal TIs for blood and myocardium, respectively[17]. Voxels in which the blood/myocardium mixture is suboptimal for a given TI appear grey as compared to the surrounding blood and endocardium. Interestingly, a black rim similar to the one in the heart could be seen in the stomach (Figure 4). Here, a signal-depleted rim at the mucosa/luminal border appeared at a certain TI. The mechanism is likely similar to that in the heart.

In theory, the black rim could also represent endocardium. In this scenario, one would postulate the optimal TI for the endocardium to be different (shorter) from that for the myocardium. At the optimal TI for myocardium, the endocardium may be difficult to discern because both the signal of the endocardium and that of blood would be greater than zero. The thin grey endocardium between the black myocardium and the grey blood may not form a strong enough contrast with the latter.

### Limitations

Although the presented case examples act as reasonably strong evidence for the concept of similar TIs in the RV and LV myocardium it would be further proven by a systematic study acquiring and comparing higher and lower resolution datasets. It would be expected that the high resolution data would show agreement in the LV and RV null times, while the lower resolution data would show an apparent difference. An animal study could also compare the true thickness of the RV myocardium with what is measured in LGE imaging at different TIs. Clinical trials in patients with pathology of the RV anterior free wall are indicated to assess the usefulness of systolic LGE imaging.

This study only relates to magnitude reconstructed late gadolinium enhanced imaging and not the phase sensitive inversion recovery (PSIR) approach that is now increasing being used. The use of (PSIR) leads to a display that is rather insensitive to TI and does not have a dark rim artefact as described (i.e., image appearance is constant across wide range of TI)[20]. Although PSIR does experience partial volume cancellation, it does not result in a dark rim artefact. In magnitude images, the cancellation results in a pixel with near zero signal intensity, and adjacent pixels that are positive and negative will both appear bright. In the PSIR case, the adjacent pixels have the correct polarity and will be negative and positive respectively, with the partial volume pixel midway. As a result, PSIR images acquired with TI too short may be retrospectively window and levelled to display a dark myocardium (nulled normal) with the adjacent blood or fat being bright, and the partial volume border midway[20]. In the RV wall, if the partial volume region has an appreciable width with respect to the wall thickness, then the net result will be that the true normal region will be very small (or there may even be no uncontaminated normal tissue). So the assessment of a thin RV wall would be degraded by poor resolution also for PSIR.

## 5. Conclusion

From our observations, we deduct that the TI for the RV is similar to that of the LV. Black rims at the interfaces between endocardium and cavity and between epicardium and epicardial fat with a thin and hardly discernable layer of myocardium in between may be misinterpreted as nulled RV myocardium. This may falsely suggest a TI that is different from optimal for the LV myocardium. In clinical practice, it may be advisable to choose the TI slightly too long rather than too short to avoid the black rim artifact that obscures RV myocardium. In-plane spatial resolution should not be lower than 1.5 mm in order to account for the thin RV free wall. Imaging in systole rather than diastole may present a superior strategy when targeting subtle and diffuse areas of LGE in the RV free wall.

## 6. Authors' contributions

LGW carried out part of the clinical studies, participated in its design and coordination, and drafted the manuscript. CM participated in the design and conceptualization of the physical background of the black rim artifact. LV carried out the computer simulations. SJY conceived of the study, participated in its design and coordination and supervised the clinical studies. All authors read and approved the final manuscript.
